# Luteinizing hormone-based modified GnRH antagonist protocol in normal responders undergoing *in vitro* fertilization treatment: A multi-center randomized controlled trial

**DOI:** 10.3389/fendo.2022.922950

**Published:** 2022-08-11

**Authors:** Shan Liu, Yasu Lv, Minghui Liu, Shuo Han, Xiaoqun Liu, Zhiming Zhao, Wei Cui, Aijun Yang, Yuan Li

**Affiliations:** ^1^ Medical Center for Human Reproduction, Beijing Chao-Yang Hospital, Capital Medical University, Beijing, China; ^2^ Center of Reproductive Medicine, Research Institute of Family Planning of Hebei Province, Shijiazhuang, China; ^3^ Department of Reproductive Medicine, The Second Hospital of Hebei Medical University, Shijiazhuang, China; ^4^ Center of Reproductive Medicine, The Second Hospital Affiliated to Shandong University of Traditional Chinese Medicine, Jinan, China; ^5^ Affiliated Hospital of Jining Medical University, Jining, China

**Keywords:** ovarian stimulation, gonadotrophin releasing hormone antagonist protocol, luteinizing hormone, reproductive outcome, *in vitro* fertilization

## Abstract

**Objective:**

To study the clinical efficacy and cost-effectiveness of a modified gonadotrophin-releasing hormone (GnRH) antagonist protocol based on luteinizing hormone (LH) levels through one complete assisted reproductive technology (ART) cycle in normal responders.

**Design:**

Non-inferiority, multicenter randomized controlled trial.

**Setting:**

University-based hospitals and an academic medical center.

**Patients:**

A total of 372 patients fulfilled the inclusion criteria and were eligible to participate.

**Intervention(s):**

Participants were randomized at a 1:1 ratio and stimulated with the conventional flexible GnRH antagonist protocol (control group) or LH-based modified GnRH antagonist protocol (study group).

**Main Outcome Measures:**

The primary outcome was the cumulative ongoing pregnancy rate per aspiration. The secondary outcomes were number of oocytes retrieved, number of good quality embryos, cumulative positive βhCG rate, cumulative clinical pregnancy rate, pregnancy loss rate, moderate and severe ovarian hyperstimulation syndrome (OHSS), and financial expenditure.

**Results:**

The cumulative ongoing pregnancy rate was 65.1% in the study group and 70.1% in the control group (odds ratio, 0.79; 95% confidence interval, 0.50–1.26; *P* = 0.33). The multivariate regression analyses results showed that the number of retrieved oocytes was positively associated with the odds for a higher cumulative ongoing pregnancy rate (adjusted odds ratio, 1.11, 95% confidence interval, 1.06–1.17, *P* < 0.001). The treatment protocol, female age, and body mass index were not independent predictors. The incremental cost-effectiveness ratio for luteinizing hormone-based gonadotrophin releasing hormone antagonist protocol *versus* the conventional flexible gonadotrophin releasing hormone antagonist protocol was estimated at 3568.6 USD for each additional ongoing pregnancy.

**Conclusion:**

The luteinizing hormone-based gonadotrophin releasing hormone antagonist protocol had clinical efficacy similar to the conventional flexible gonadotrophin releasing hormone antagonist protocol in normal responders undergoing *in vitro* fertilization treatment but was more cost-effective considering the cumulative ongoing pregnancy rate in the entire assisted reproductive technology cycle.

**Clinical Trial Registration:**

www.chictr.org.cn, identifier: ChiCTR1800018077

**URL of the registration site:**

http://www.chictr.org.cn/edit.aspx?pid=27389&htm=4.

**Trial registration date:**

29 August 2018.

**Date of first patient enrollment:**

1 September 2018.

## Introduction

Assisted reproductive technology (ART) is the most important method to treat infertility. Gonadotrophin releasing hormone (GnRH) antagonist protocol has been widely used in the past few decades. The conventional GnRH antagonist protocols include fixed and flexible protocols, with the antagonist administrated from day 5 or 6 onwards, or when the dominant follicle had reached 14 mm, respectively (1, 2). Since its introduction, many studies have sought to determine the best day to start GnRH antagonist administration and the optimal daily dosage (3, 4).

The GnRH antagonist administration aims to prevent an untimely luteinizing hormone (LH) surge and premature luteinization. Previous studies evaluating technical aspects of these protocols to guide antagonist administration mainly focused on the day of gonadotropin stimulation or the diameter of the follicles rather than the LH level. In recent years, increasing attention has been paid to the role of LH. The secretion and response of LH levels to the GnRH antagonists vary widely between individuals (5–8). Previous studies have indicated that an ultra-high or low LH level would cause harm to pregnancy outcomes (9–11). Although it has remained controversial, an LH stimulation threshold is required for adequate follicular development and oocyte maturation. This made us think whether LH could be used as an indicator for the timing of antagonist addition, and a modified protocol named “LH-based GnRH antagonist protocol” was proposed. Our previous proof-of-concept study showed that this protocol provided comparable results to the traditional flexible antagonist protocol (12). The results proved that LH levels may be used as an indicator for the time of antagonist addition. However, it had inherent limitations because of its nature of retrospective design. The inclusion criteria were kind of too general and needed to be further refined. On the other hand, assessment of the cost-effectiveness of treatment is essential before recommendations can be made. To further confirm the validity of our previous observations and evaluate its clinical efficacy, we performed this non-inferiority randomized controlled trial (RCT) in normal responders undergoing *in vitro* fertilization (IVF). Besides, cost-effectiveness analysis was also performed to provide reference for medical decision-making.

## Materials and methods

### Study design and patients

This trial was a non-blinded, multicenter RCT. It was approved by the Ethics Committee of Beijing Chao-Yang Hospital, Capital Medical University (number 2018-SCI-194), and the institutional review boards of all the participating centers. This trial was registered at the Chinese Clinical Trial Registry (number ChiCTR1800018077, registered in August 2018), and the protocol has previously been published (13). All participating couples provided written informed consent. Inclusion criteria were as follows: (1) aged 23–38 years; (2) a spontaneous menstrual cycle length of 21–35 days; (3) an antral follicle count (AFC) of 8–20; (4) a body mass index (BMI) of 18–28 kg/m^2^. The exclusion criteria included the following: (1) recurrent spontaneous abortion; (2) a diagnosis of polycystic ovarian syndrome (PCOS); (3) uterine abnormalities such as submucosal myoma, adenomyosis, or intrauterine adhesion; (4) a chronic medical disease affecting pregnancy outcomes such as diabetes mellitus or uncontrolled hypertension.

### Randomization and masking

Randomization was performed by a clinician on the initial day of ovarian stimulation. Patients were randomized at a 1:1 ratio using a web-based concealed randomization code generated by a central online database (www.medresman.org). After randomization, patients were assigned into two groups: a control group stimulated with the conventional flexible GnRH antagonist protocol or a study group stimulated with the LH-based GnRH antagonist protocol. Clinicians and patients were not masked for the assigned interventions.

### Treatment

150-300 IU recombinant follicle-stimulating hormone (r-FSH, Gonal F, Merck Serono, Darmstadt, Germany) was administered daily for four to five days from Day 2-3 of the menstrual cycle. Hormone tests and ultrasonographic monitoring were performed 4-5 times at regular intervals during ovarian stimulation, as follows: 1) day 1 of stimulation (early follicular phase); 2) 4-5 days after stimulation initiation (mid-follicular phase); 3) 2 days later, i.e., 6-7 days after initiation (mid-late follicular phase); 4) the day of triggering (late follicular phase). Then gonadotropin dose might be adjusted according to the hormone levels and follicular development.

For patients in the study group, administration of the GnRH antagonist (Cetrotide, Merck Serono) was based on the LH level from day 6 of ovarian stimulation, as mentioned before (14). Briefly, no antagonist was administered if LH level was ≤4 IU/L; 0.125 mg antagonist was administered daily for two days, until the next blood test, if 4 < LH level ≤ 6 IU/L; 0.25 mg antagonist was administered daily for two days if 6 < LH level ≤ 10 IU/L; 0.375 mg antagonist was administered for one day if 10 < LH level ≤ 15 IU/L; 0.5 mg antagonist was administered for one day if LH level >15 IU/L. The decision to continue antagonist administration was based on whether the subsequent LH test result was > 4 IU/L. This procedure continued until trigger day. Patients in the control group were administered a flexible GnRH antagonist protocol. They received 0.25 mg antagonist daily until trigger day if at least one follicle had reached a diameter of 14 mm.

For both protocols, when more than two follicles were ≥18 mm in diameter, 0.2 mg triptorelin (Decapeptyl, Ipsen, Paris, France) and 2,000–3,000 IU human chorionic gonadotropin (hCG) were administered. Ovum pick up (OPU) was performed 36 h later, and the oocytes were fertilized by IVF or intracytoplasmic sperm injection (ICSI). The following treatment, including fresh and frozen embryo transfer, were underwent according to the clinical standards of the participating centers. The policies were described in detail elsewhere previously (14). Luteal-phase support was started from the day of oocyte retrieval with 90 mg daily of 8% vaginal progesterone gel (Crinone, Merck Serono) and 10 mg twice daily oral dydrogesterone (Duphaston, Abbott Biologicals B.V., Olst, the Netherlands). A maximum of two good-quality cleavage embryos were transferred on D3. All surplus embryos were cultured for two or three more days, and good-quality blastocysts were vitrified. If pregnancy was achieved, the luteal phase support was continued until 10 weeks’ gestation after fresh embryo transfer. The endometrium was prepared for fresh and frozen embryo transfer (FET) cycles by either a natural or an artificial cycle regimen. Following a natural cycle regimen, luteal phase support with 10 mg dydrogesterone twice daily from ovulation day until 7 weeks’ gestation was administered. For the artificial cycle regimen, 6 mg oral estradiol valerate (Progynova, Bayer AG, Leverkusen, Germany) daily was initiated from day 2–3 of the menstrual cycle for 10–14 days. If the endometrium was ≥7 mm, 90 mg vaginal progesterone gel daily and 10 mg dydrogesterone twice daily were added. Oral estradiol valerate was continued until 8 weeks’ gestation, and vaginal progesterone gel and dydrogesterone were continued until 12 weeks’ gestation.

### Reproductive outcomes

End-of-study was the achievement of an ongoing pregnancy or transfer of all derived embryos through a complete ART cycle, including the fresh and FET cycles. The primary outcome was the cumulative ongoing pregnancy rate per aspiration. The secondary outcomes were number of oocytes retrieved, number of good quality embryos, cumulative positive βhCG rate, cumulative clinical pregnancy rate, pregnancy loss rate, moderate and severe ovarian hyperstimulation syndrome (OHSS), and financial expenditure. Ongoing pregnancy was defined as visible fetal heart activity on ultrasonography from 12 weeks of gestation onwards. Positive βhCG was defined as plasma βhCG >10 IU/L. Clinical pregnancy was confirmed by observing a gestational sac by ultrasonography 2-3 weeks later.

### Sample size

A retrospective assessment of our clinical database found a cumulative ongoing pregnancy rate per aspiration in normal responders of approximately 70%. Considering that a non-inferiority threshold should retain 80% of the conventional antagonist protocol clinical effect, a minimal clinical important difference of 14% was adopted. With a one-sided alpha of 2.5% and statistical power of 80%, 338 patients were needed. Considering a dropout rate of 10%, 372 patients were required.

### Statistical analysis

**Figure 1:**
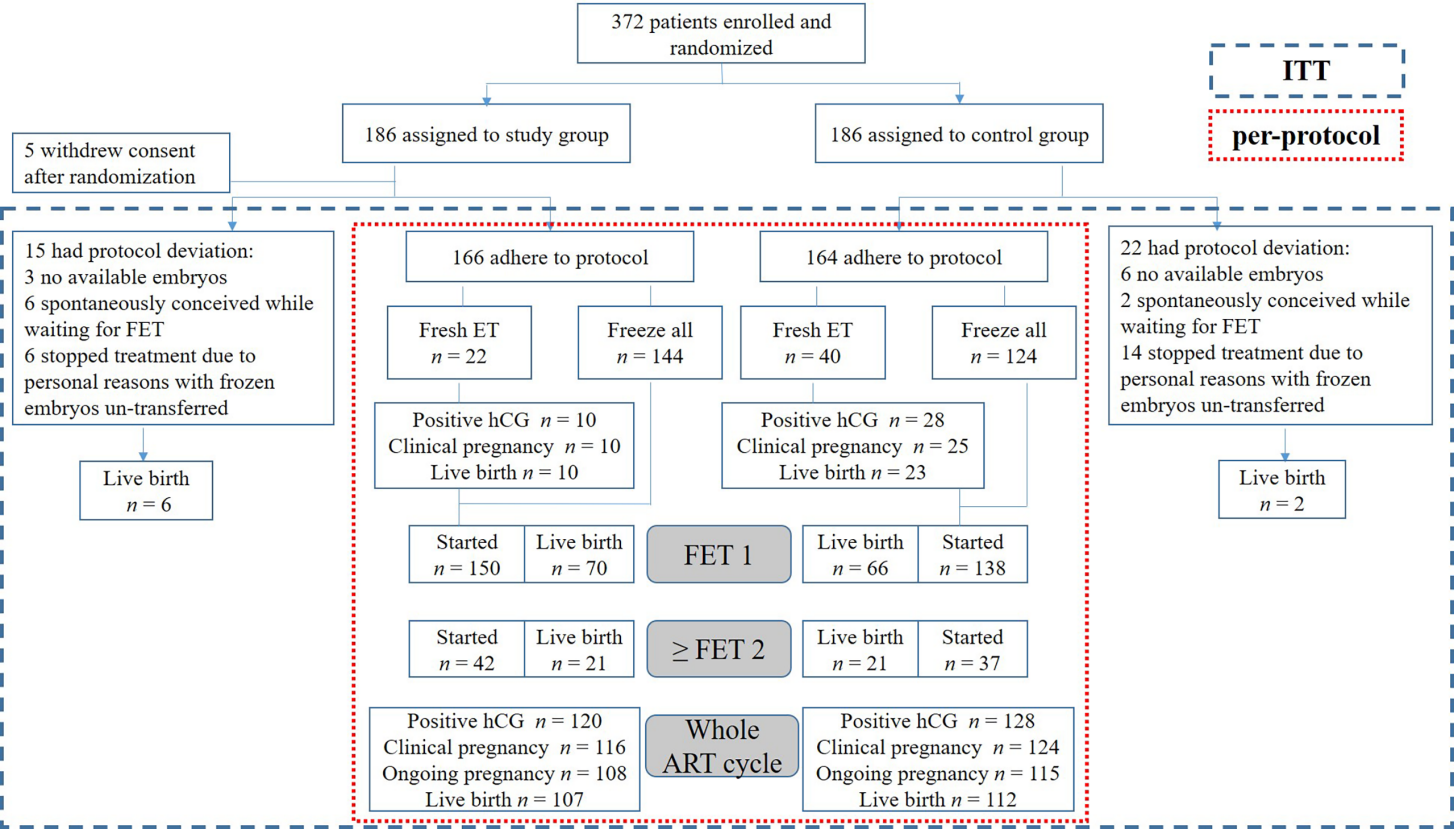
Trial flow chart. An overview of the study process and cumulative reproductive outcomes in the study and control groups. ITT, intention-to-treat; ET, embryo transfer; FET, frozen embryo transfer; hCG, human chorionic gonadotropin; ART, assisted reproductive technology.

A per-protocol comparison of the primary outcome was performed among those who adhered to their respective protocol. Intention-to-treat analysis was applied to the 367 eligible patients who did not withdraw their consent (**Figure 1**). Continuous data are expressed as means ± standard deviations. Between-group differences were tested by an independent samples *t*-test for normally distributed data and Wilcoxon rank-sum test for not normally distributed data. Categorical data are presented as frequency and percentage; differences were assessed by the chi-squared test or Fisher’s exact test.

The cumulative ongoing pregnancy rate per aspiration was assessed crudely and using multivariate logistic regression analysis. The decision to add each potential confounding factor to the model was based on previous scientific evidence and the results in the unadjusted analyses. These factors included age, BMI, the number of oocytes retrieved, mean LH level during ovarian stimulation, and LH level on the triggering day. All analyses were done with the IBM SPSS Statistics for Windows, Version 22.0 (IBM Corp., Armonk, NY, USA). A *P*-value < 0.05 was considered statistically significant.

### Cost-effectiveness analysis

Economic evaluation in this study focused only on pharmacological compounds costs during ovarian stimulation till trigger day. The cost was equal to the unit cost of the drug multiplied by the total quantity administered. Costs were based on Beijing General Hospital prices and calculated in CNY and then converted to USD (1 CNY=0.1564 USD). We calculated the mean costs and effectiveness for both groups, with the conventional flexible GnRH antagonist protocol acting as the reference.

The incremental cost-effectiveness ratio (ICER) was based on the incremental cost per patient and cumulative ongoing pregnancy rate of the LH-based protocol compared to the flexible antagonist protocol. The incremental cost per patient reflected the additional cost per patient undergoing the LH-based antagonist protocol rather than the conventional protocol. The incremental cumulative ongoing pregnancy rate reflected the change in the cumulative ongoing pregnancy rate when the LH-based antagonist protocol was used instead of the conventional protocol. The ICER will be estimated as the ratio between difference in costs between protocols and the difference in cumulative ongoing pregnancy rates.

## Results

Recruitment was done between September 2018 and June 2020. Patients were followed until all embryos obtained from an entire ART cycle were used or an ongoing pregnancy was achieved. Those who spontaneously conceived while waiting for FET and stopped treatment due to personal reasons with frozen embryos un-transferred for more than 12 months were exclude from the per-protocol analyses. **Figure 1** displays the patient enrollment flowchart. The 372 enrolled patients were randomly assigned to either study or control group. Five patients in the study group withdrew their consent after randomization and were defined as dropped out cases. The baseline demographic and clinical characteristics in the two groups were compared, as shown in **Table 1**.

**Table 1:** Baseline characteristics of the intention-to-treat population.

	Study group *n* = 181	Control group *n* = 186
**Age (year)**	31.40 ± 3.51	31.61 ± 3.08
**BMI (kg/m^2^)**	22.38 ± 3.14	22.33 ± 3.12
**Duration of infertility (year)**	2.92 ± 1.90	2.71 ± 2.02
**Diagnosis**		
** Primary infertility** ** Secondary infertility**	121 (66.85%) 60 (33.15%)	128 (68.82%) 58 (31.18%)
**Primary diagnosis of infertility**		
** Tubal factor** ** Anovulation** ** Endometriosis** ** Male factor** ** Combined factors**	97 (53.59%) 12 (6.63%) 6 (3.31%) 18 (9.94%) 48 (26.52%)	135 (72.58%) 7 (3.76%) 0 (0.00%) 10 (5.38%) 34 (18.28%)
**Basal FSH (IU/L)**	6.85 ± 1.87	7.05 ± 2.15
**Basal LH (IU/L)**	4.57 ± 1.87	4.57 ± 1.97
**Basal E_2_ (pg/mL)**	45.54 ± 15.49	45.67 ± 15.02
**AFC**	15.80 ± 5.67	15.67 ± 4.72

Values are represented as mean ± standard deviation, or n (%). BMI, body mass index; FSH, follicle-stimulating hormone; LH, luteinizing hormone; AFC, antral follicle count; E_2_, estradiol; Study group: LH-based GnRH antagonist protocol; Control group: conventional flexible GnRH antagonist protocol.

### Stimulation outcomes

Patients in the study (*n* = 166) and control (*n* = 164) groups who adhered to their respective treatments were included in the following per-protocol analyses. As shown in **Table 2**, many ovarian stimulation characteristics were similar in the two groups; however, LH level on the trigger day was considerably higher in the study group (2.81 ± 1.89 vs. 2.14 ± 1.31 IU/L, *P* < 0.001). Hormone tests were performed regularly during ovarian stimulation, representing the early, mid, and late follicular phases. Then mean LH level could be figured out, which was much higher in the study group (3.64 ± 1.63 vs. 3.29 ± 1.27 IU/L, *P* = 0.032). The study group had a lower administered doses of GnRH antagonist (0.38 ± 0.27 vs. 1.01 ± 0.30 IU/L, *P* < 0.001) and less recombinant LH (rLH) (63.04 ± 44.63 vs. 124.39 ± 77.49 IU, *P* < 0.001) was used to maintain a reasonable LH level and normal follicular development. Similar ovarian response indexes, including follicle output rate (FORT), follicle-to-oocyte index (FOI), and ovarian sensitivity index (OSI), were noted in both groups, as were laboratory outcomes. Although similar total gonadotropin (Gn) dose was delivered in both groups, with lower administered doses of GnRH antagonist and rLH in the study group, it had considerably lower financial expenditure during ovarian stimulation (1,031.51 ± 281.59 vs. 1,209.94 ± 307.12 USD, *P* < 0.001). Ovarian stimulation and laboratory outcomes were also analyzed by intention-to-treat and showed similar results (data not shown).

**Table 2:** The per-protocol population ovarian stimulation and laboratory outcomes.

Group	Study group	Control group	*P*-value
***n***	166	164	
**Total Gn dose (IU)**	1984.19 ± 582.33	2018.71 ± 536.63	0.58
**Duration of Gn stimulation**	9.58 ± 1.34	9.51 ± 1.09	0.59
**LH on triggering day (IU/L)**	2.81 ± 1.89	2.14 ± 1.31	<0.001
**E_2_ on triggering day (pg/mL)**	3345.35 ± 1732.89	3211.84 ± 1448.31	0.74
**P on triggering day (ng/mL)**	0.83 ± 0.47	0.76 ± 0.34	0.50
**Endometrial thickness on triggering day (mm)**	10.69 ± 2.13	10.47 ± 2.25	0.37
**Mean LH level during stimulation (IU/L)**	3.64 ± 1.63	3.29 ± 1.27	0.03
**GnRH antagonist dose (mg)**	0.38 ± 0.27	1.01 ± 0.30	<0.001
**rLH dose (IU)**	63.04 ± 44.63	124.39 ± 77.49	<0.001
**FORT (%)**	0.81 ± 0.29	0.82 ± 0.33	0.87
**FOI (%)**	0.93 ± 0.41	0.98 ± 0.40	0.30
**OSI**	7.95 ± 4.68	7.90 ± 3.86	0.61
**No. of oocytes retrieved**	14.26 ± 6.27	14.68 ± 5.85	0.53
**No. of 2PN**	8.73 ± 5.36	8.84 ± 4.27	0.35
**No. of high-quality embryos on D3**	3.85 ± 3.14	4.03 ± 2.99	0.31
**Financial expenditure (USD)**	1,031.51 ± 281.59	1,209.94 ± 307.12	<0.001

Values are presented as mean ± standard deviation.

P-values were calculated using the chi-squared or Fisher’s exact test for categorical data and the t-test for continuous data.

FORT, follicular output rate = No. of pre-ovulatory follicles/AFC; FOI, follicle-to-oocyte index = No. of oocytes retrieved/AFC; OSI, ovarian sensitivity index = number of retrieved oocytes/total Gn dose × 1,000; Gn, gonadotropin; GnRH, gonadotropin releasing hormone; LH, luteinizing hormone; rLH, recombinant luteinizing hormone; E_2_, estradiol; P, progesterone; 2PN, two pronuclei; D3, Day 3.

Study group: LH-based GnRH antagonist protocol; Control group: conventional flexible GnRH antagonist protocol.

### Reproductive outcomes

The primary outcome, cumulative ongoing pregnancy rate per ART cycle, was 65.1% in the study group and 70.1% in the control group (odds ratio [OR], 0.79; 95% confidence interval [CI], 0.50–1.26; *P* = 0.33; **Table 3**), meeting the predefined non-inferiority objective. Fresh embryo transfer was performed in 22 patients in the study and 40 in the control group. Other patients were performed “freeze-all strategy” and underwent FET later. The secondary outcomes were comparable between the two groups for the fresh embryo transfer and first FET cycles in patients undergoing “freeze-all strategy”. Cumulatively, the two groups had similar reproductive outcomes for the entire ART cycle, including the pregnancy loss rates. No severe treatment-emergent adverse events were reported, and no cycle was canceled due to excessive ovarian response or unexpected premature ovulation.

**Table 3:** Reproductive outcome analysis.

	Study group	Control group	OR (95% CI)	*P*-value
**First embryo transfer cycle**				
** *n***	166	164		
** Positive ßhCG, *n* (%)**	89 (53.6%)	104 (63.4%)	0.67 (0.43–1.04)	0.07
** Clinical pregnancy, *n* (%)**	81 (48.8%)	96 (58.5%)	0.67 (0.44–1.04)	0.08
** Ongoing pregnancy, n (%)**	76 (45.8%)	86 (52.4%)	0.77 (0.50–1.18)	0.23
** Live birth, n (%)**	75 (45.2%)	84 (51.2%)	0.78 (0.51–1.21)	0.27
**Fresh ET cycle**				
** *n***	22	40		
** Positive ßhCG, n (%)**	10 (45.5%)	28 (70.0%)	0.36 (0.12–1.05)	0.10
** Clinical pregnancy, *n* (%)**	10 (45.5%)	25 (62.5%)	0.5 (0.17–1.44)	0.29
** Ongoing pregnancy, n (%)**	10 (45.5%)	23 (57.5%)	0.62 (0.22–1.76)	0.43
** Live birth, n (%)**	10 (45.5%)	23 (57.5%)	0.62 (0.22–1.76)	0.43
**First FET cycle of the “freeze-all” patients**				
** *n***	144	124		
** Positive ßhCG, n (%)**	81 (56.3%)	77 (62.1%)	0.78 (0.48–1.28)	0.38
** Clinical pregnancy, *n* (%)**	73 (50.7%)	73 (58.9%)	0.72 (0.44–1.17)	0.22
** Ongoing pregnancy, n (%)**	67 (46.5%)	63 (50.8%)	0.84 (0.52–1.36)	0.54
** Live birth, n (%)**	66 (45.8%)	61 (49.2%)	0.87 (0.54–1.41)	0.62
**Whole ART cycle**				
** *n***	166	164		
** Cumulative positive ßhCG, *n* (%)**	120 (72.3%)	128 (78.0%)	0.73 (0.44–1.21)	0.23
** Cumulative clinical pregnancy, *n* (%)**	116 (69.9%)	124 (75.6%)	0.75 (0.46–1.22)	0.24
** Cumulative ongoing pregnancy, *n* (%)**	108 (65.1%)	115 (70.1%)	0.79 (0.50–1.26)	0.33
** Cumulative live birth, *n* (%)**	107 (64.5%)	112 (68.3%)	0.84 (0.53–1.33)	0.46
** Singleton**	79	83		
** Twins**	28	29		
**Pregnancy loss**				
** Biochemical miscarriage, *n* (%)**	4 (3.5%)	4 (3.1%)	1.13 (0.28–4.61)	0.87
**Clinical pregnancy loss**				
** First trimester pregnancy loss, *n* (%)**	8 (7.3%)	9 (7.3%)	1.00 (0.37–2.69)	0.99
** Second trimester pregnancy loss, *n* (%)**	1 (1.0%)	3 (2.6%)	0.37 (0.04–3.61)	0.37
**OHSS**				
** Moderate**	0	1	–	–
** Severe**	0	0	–	–
**Canceled cycles, *n***	0	0	–	–

For the first embryo transfer cycle, fresh embryos were transferred in 22 and 40 patients in the study group and control group, respectively. 144 patients in the study group and 124 in the control group were performed “freeze-all strategy” and underwent FET later. OR, odds ratio; CI, confidence interval; ßhCG, beta human chorionic gonadotropin; ET, embryo transfer; FET, frozen embryo transfer; OHSS: ovarian hyperstimulation syndrome.

**Table 4** summarizes the results of univariate and multivariate regression analyses for the cumulative ongoing pregnancy rate in the two groups. The results showed that the number of retrieved oocytes was associated with increased odds for a higher cumulative ongoing pregnancy (adjusted OR, 1.11; 95% CI, 1.06–1.17; *P* < 0.001). Treatment protocol, female age, and BMI were not independent predictors. The association between mean LH level during stimulation and cumulative ongoing pregnancy was statistically insignificant (OR, 1.07; 95% CI, 0.89–1.29; *P* = 0.477). Similar results were shown for LH level on trigger day.

**Table 4:** Crude and adjusted OR for cumulative ongoing pregnancy rate of the per-protocol patients.

Exposure	OR	95% CI	*P*-value	adj. OR	95% CI	*P*-value
		Univariate			Multivariate	
Protocol						
Flexible GnRH antagonist protocol	1			1		
LH-based GnRH antagonist protocol	0.79	0.50–1.26	0.33	0.71	0.44–1.16	0.18
Age (years)						
<35	1			1		
≥35	0.67	0.38–1.17	0.16	0.80	0.44–1.45	0.46
BMI (kg/m^2^)						
<24	1			1		
24-28	0.73	0.43–1.24	0.25	0.67	0.38–1.18	0.17
>28	0.57	0.23–1.43	0.23	0.62	0.23–1.64	0.33
No. of oocytes retrieved	1.12	1.07–1.17	<0.001	1.11	1.06–1.17	<0.001
Mean LH level during ovarian stimulation	1.04	0.89–1.22	0.61	1.07	0.89–1.29	0.48
LH on triggering day	0.87	0.76–0.99	0.04	0.93	0.79–1.09	0.34

OR, odds ratio; adj. OR: adjusted odds ratio; CI, confidence interval, GnRH, gonadotropin releasing hormone; LH, luteinizing hormone; BMI, body mass index.

### Cost-effectiveness analysis

The mean pharmacological compounds cost per patient undergoing ovarian stimulation with the LH-based protocol was 1,031.51 USD, and was 1,209.94 USD for the control protocol. The cost per cumulative ongoing pregnancy was 1,584.50 USD for the study protocol *versus* 1,726.02 USD for the control protocol. This study estimated the ICER for the LH-based GnRH antagonist protocol *versus* the conventional flexible GnRH antagonist protocol at 3,568.6 USD for each additional ongoing pregnancy (**Table 5**).

**Table 5:** Cost-effectiveness analysis of the LH-based and conventional flexible GnRH antagonist protocols.

Protocol	Costs (USD)	Effectiveness	C/E	ICER (USD)
**Conventional flexible GnRH antagonist protocol**	1209.94	0.70	1726.02	—
**LH-based GnRH antagonist protocol**	1031.51	0.65	1584.50	3568.60

LH, luteinizing hormone; GnRH, gonadotropin releasing hormone; C/E, costs/effectiveness; ICER, incremental cost-effectiveness ratio.

## Discussion

This study was the first RCT to investigate the clinical efficacy and cost-effectiveness of the LH-based modified GnRH antagonist protocol during ovarian stimulation in normal responders. We found that the reproductive outcomes were comparable, i.e., the LH-based protocol was not inferior in clinical efficacy. Moreover, it was more cost-effective considering the cumulative ongoing pregnancy rate in the entire ART cycle.

LH has a significant impact on morphological and functional changes of the oocyte and determines its meiotic status and ability to be fertilized (15). LH levels vary between individuals during ovarian stimulation. Recent evidence showed that LH glycosylation variants, genetic variants of LH and its receptor, and female age could negatively affect gonadotropin action and ovarian response (7, 8, 16). GnRH, estradiol, and anti-Mullerian hormone (AMH) are all potential factors implicated in the control of LH level (17, 18). Our previous proof-of-concept study demonstrated that patients with sustained low LH levels might not require antagonist administration during ovarian stimulation. If administered, the antagonist might further decrease the LH level and adversely affect reproductive outcomes (12). Besides, we found in another previous study that the cumulative live birth rate (CLBR) in the low LH group was significantly lower than in the high LH group. Patients with low LH levels had a lower live birth rate (LBR) after fresh embryo transfer but comparable LBR after the first FET in freeze-all cycles (19). On the other hand, high LH levels during the follicular phase were also associated with poor oocyte or embryo quality and impaired endometrial receptivity and, consequently, with a negative impact on reproductive outcomes (20, 21). Thus, we believed that the use of GnRH antagonists as LH level regulators should take the LH level during ovarian stimulation into consideration for a better individualized stimulation protocol. Regular hormone tests could conveniently monitor the LH level. The GnRH antagonist action is dose-dependent, so its flexible addition could maintain the LH within the desired range. The dosage of the antagonist was significantly reduced in our study group, with no cycle cancellation due to premature ovulation.

A single serum LH measurement on a predefined day cannot reflect the average LH concentration during the follicular phase. This was one of the reasons for the controversial results reported in previous studies. Chen et al. measured LH level at a mean interval of 2.3 days across the follicular phase to demonstrate the low area under the curve for serum LH and obtain a cut-off value (22). In the present study, 4–5 hormone tests were performed at regular intervals for an average stimulation duration of 8–10 days. We believed these tests could optimally reflect the LH changes throughout the cycle, even though they were not measured daily. The mean LH levels during stimulation was compared between the two groups. Although the independent samples *t*-test indicated between-group differences, multivariate regression analyses did not demonstrate the influence on the cumulative ongoing pregnancy rates (**Tables 2**, **4**). One possible explanation was that patients were randomly assigned to the study and control groups rather than grouped according to their LH levels. Although the mean LH level in the control group was lower, this did not mean that all patients in the control group had low LH. Besides, rLH was supplemented in both groups to benefit patients with LH deficiency. With a lower GnRH antagonist dosage and a relatively high mean LH level, the rLH dose in the study group was much lower than in the control group. Thus, the economical expenditure of the patients was reduced.

Many indicators have been raised to evaluate the ovarian response during stimulation, including FORT, FOI, and OSI. Each of them has advantages and disadvantages. FORT assesses only the number of follicles before and after stimulation, but does not consider the degree of ovarian stimulation, i.e., the Gn dosage, or assess the number of oocytes retrieved, which is closely related to live birth. FOI could be influenced by the initial Gn dosage, genetic or environmental factors, asynchronous follicular development, and technical issues (23). The OSI does not consider the Gn type (e.g., whether LH or LH analog was added) or the AFC (24). In the present study, we statistically analyzed all three indicators to provide a more comprehensive assessment of ovarian response. The results showed that the two groups were comparable, further suggesting that the LH-based GnRH antagonist protocol was not inferior to the conventional flexible GnRH protocol in terms of ovarian response.

Recent literature suggested that clinical efficacy should be accompanied by economic studies. Even when the primary outcome was similar (as was the case in this study), cost-effectiveness analysis should be performed to assess the effectiveness rather than the efficacy of the procedure (25). Both direct and indirect costs should be included when calculating the cost of IVF. Direct non-medical costs (e.g., travel and accommodation costs) and indirect costs (e.g., income lost) data were intangible and difficult to calculate. Furthermore, these costs had nothing to do with the difference in stimulation protocol. The differences between the groups in the present study were limited to the time and dose of the antagonist administration. Other direct medical costs, such as the number of follow-up visits and examinations, were similar and, therefore, excluded from the analysis. There was no severe OHSS requiring treatment in either group. Taken together, the economic evaluation in this study focused only on drug costs during ovarian stimulation till trigger. The results demonstrated that the LH-based GnRH antagonist protocol was more cost-effective than the conventional protocol. It would be particularly important for patients in the developing countries without public health insurance coverage of treatments for infertility.

The present study has several limitations. First, we included a relatively selected population of females expected to show normal ovarian response. Hence, whether these results could be extrapolated to the general population requires further investigation. One more topic-related RCT (number ChiCTR1800018129) in which PCOS patients are enrolled is currently under way. Second, the LH cut-off values for the various GnRH antagonist doses were based on our previous study and clinical observations. However, we cannot affirm that they are the best discriminatory thresholds. Third, this study had a multi-center setting with subjects from the participating centers competing for inclusion. Most subjects were recruited from the Medical Center for Human Reproduction, Beijing Chao-Yang Hospital. Therefore, analyses considering differences among the participating centers were not performed. The representativeness of the study population was limited, affecting the ability to extrapolate the study findings. Fourth, the proportion of fresh embryo transfer cycles was relatively low in the present study, as clinicians tended to follow the freeze-all strategy. For future research, it would be worthwhile to collect more clinical data on fresh embryo transfer cycles to further demonstrate the mechanism of LH levels during ovarian stimulation on follicle development and endometria receptivity. And on this basis, it would provide more robust evidence in selection of the most appropriate cycle for fresh embryo transfer, and provide indications for individualized treatment and freeze-all strategy.

## Conclusion

The LH-based GnRH antagonist protocol was not inferior to the conventional flexible GnRH antagonist protocol in clinical efficacy for normal responders. However, it was more cost-effective, considering the cumulative ongoing pregnancy rate in the entire ART cycle. Further large scale RCTs are needed to see if this protocol can be applied to the entire population undergoing IVF.

## Data availability statement

The original contributions presented in the study are included in the article/supplementary material. Further inquiries can be directed to the corresponding author.

## Ethics statement

The studies involving human participants were reviewed and approved by Ethics Committee of Beijing Chao-Yang Hospital, Capital Medical University. The patients/participants provided their written informed consent to participate in this study.

## Author contributions

SL: data collection and statistical analysis; drafting of the manuscript. YSL, ML, SH, XL, ZZ, WC, and AY: patient’s treatment and revising of the manuscript. YL: supervision of the study concept and review of manuscript. All authors performed revision of intellectual content of the final version of the article.

## Funding

This study was funded by the 1351 Talent training Program of Beijing Chao-Yang Hospital (CYXX-2017-20); Capital Health Development Scientific Research Project (Independent Innovation, 2020-1-2039); Beijing Health Promotion Foundation (2019-09-05); 2020 Fertility Research Program of Young and Middle-aged Physicians-China Health Promotion Foundation; Beijing Hospitals Authority Youth Programme (QML20200301).

## Acknowledgments

The authors thank all the patients participated in this study, as well as the clinicians, embryologists, and nursing staff in all the participating centers: Medical Center for Human Reproduction, Beijing Chao-Yang Hospital, Capital Medical University; Research Institute of Family Planning of Hebei Province; Department of Reproductive Medicine, The Second Hospital of Hebei Medical University; Center of Reproductive Medicine, The Second Hospital Affiliated to Shandong University of Traditional Chinese Medicine; Affiliated hospital of Jining Medical University.

## Conflict of interest

The authors declare that the research was conducted in the absence of any commercial or financial relationships that could be construed as a potential conflict of interest.

## Publisher’s note

All claims expressed in this article are solely those of the authors and do not necessarily represent those of their affiliated organizations, or those of the publisher, the editors and the reviewers. Any product that may be evaluated in this article, or claim that may be made by its manufacturer, is not guaranteed or endorsed by the publisher.
